# Address of President J. B. Cowan, M. D., Read before the Tri-State Medical Association, Chattanooga, Tennessee, Oct. 15, 1890

**Published:** 1890-11

**Authors:** 


### ADDRESS OF PRESIDENT J. B COWAN, M. D., READ BEFORE THE TRI-STATE MEDICAL ASSOCIATION, CHATTANOOGA, TENNESSEE, OCT. 15, 1890.

*Ladies and Gentlemen and Members of the State Medical Association:*

I confess to some intimidation in the position that I occupy to-night. The avocations of a busy life have left me but little time to prepare for the duty that devolves on me at this hour. Nevertheless, I am assured, even as I stand in the presence of this large audience, and in the presence of so large a representation of medical talent, by the reflection that the cause which I come to plead is dear to us all alike, and that the feeling of interest in our great profession about which I shall attempt to talk is the same motive which assembles you here.

For we come to-day to greet each other after another year of toil; to tell of our battles fought and victories won; to speak to each other and exchange views on the great questions and principles that apply to our vocation; to talk of the mighty achievements and rapid progress of our great profession; to compare notes and stimulate each other for grander achievements for the future. Surely, then, I can rely with perfect confidence upon the sympathy and indulgence of my audience, believing that the same motive which causes me to speak will also induce you to hear. I feel confident that I may claim for my subject, also, the sympathy and attention of this intelligent audience.

For I am to talk to you of the doctor, of his preparation, his education, in its purest and noblest sense; education in the sense the wise and thoughtful of all ages have held to be indispensable to the success of all men who enter the learned professions.

The doctor is a subject that will at least interest a part of my audience. In fact, it is a subject that every individual in every community should feel an abiding interest in.

The doctor is an indispensable factor in every community.

This being the fact, there is no need of argument to prove that this indispensable factor should be a man equal to the importance and responsibility of his station.

We have often heard it said that a man was born a leader, or born to rule. However true this may be, the fact has long since been demonstrated that men are not born doctors.

There are so many prerequisites to be considered before a man can be a doctor, that we shall only consider a few of them, just enough to give some idea of the great work required to fit men for this honorable profession. The first question, then, is, what does it take to make a doctor?

The man that starts out to be a doctor must understand that it is a life of toil. There is no flowery way to the royal degree of excellence; the laggard, the indolent, the careless or the sluggard never enter the temple of fame; he never tastes of the royal feast spread for the earnest, industrious worker; he knows nothing of the grandeur of mental comprehension of the real medical scholar.

The doctor must be educated, not as that term seems to be understood in these latter days. Modern education, I fear, is too much a process of stuffing, or cramming. The word *educo* means more than this; educate means to draw out, to enlarge, to expand, to develope, to strengthen.

The development of the mental powers is like training an athlete. You would not take a boy, if you wanted to make an athlete of him, and simply stuff or cram him with the wonderful athletic feats done by others. You might do this until doom's-day without making an athlete of him. You would take him to the gymnasium and commence him with the use of the dumb-bells, the spring-board, the cross-bar, etc., and gradually develop every muscle and fibre of his body, until he had strength and endurance and activity. The result of this development or education of his muscular system would be that he would be able, with grace and elegance and ease, to perform the most difficult athletic feats. Why? Because in the education he had developed muscular ability. So in the education of the mental faculties, we are not to stuff or cram the memory full of the thoughts of other men, but we are to train the mental powers so that there shall be ability to think, and to think clearly, and to think with force, and to think correctly.

It will be seen, then, that I use the word educate in its literal and most complete sense.

We are aware of the fact that every man has within himself a certain amount of natural endowments and susceptibilities. Education is the brining out and development of these—the leading forth and organizing these forces. It is not mere scholarship. A man may be a scholar and yet be poorly educated. It is not mere learning. A man may be learned and yet unable to use either himself or his learning. It is not mere knowledge, or the mass of information which a man possesses; it is the development of his mental powers in every direction and in every aspect which constitutes true education.

And just as this development of the physical man by the education of his muscular system gives him the use of himself physically, so true mental education gives him the use of himself mentally, and makes him a power in the world and master of himself. True education leads to self-poise, self-control.

It has been truly said that the truly educated man can always command and use his faculties for all that they are worth. He may be a man of much or little capacity, accordingly as nature has made him. He may be a man of much or little power, but if he is truly educated, he will count for all he is worth. He will fulfil his destiny; he will do his part in the great work of professional life. This mental training, or education should be closely attended to before men attempt to enter the learned profession of medicine. This preparation is needful that the student may be able to grasp and master the profound philosophy and critical science of the medical profession. We need not expect an untrained or an undisciplined mind to take hold on and digest the great truths and the profound philosophy of so learned a science.

It requires systematic thought; it requires a trained mind, to grapple successfully with the occult principles of life, and then comprehend the multiple shades of departure from health into the innumerable morbid or pathological conditions to which this human organism is subject. The various sciences that are embraced under the general head of medical science demand that the doctor shall, in his education, reach out and take in a thorough knowledge and become acquainted with all these varied departments, not simply a passing acquaintance, but he must be intimately acquainted, thoroughly familiar with them. In fact, to be a doctor he must be master of these various departments of medical science. The doctor must be an anatomist, he must have the foundation upon which to build the superstructure; he must understand the geography of the human organism, not in its general outlines, but in its minutia. He must be intimately acquainted with the various tissues, organs, apparatus and systems of the body, and then he must understand the relation of one part with another, the means by which the whole is brought into one homogenous organism; the circulaticn of the blood; the nervous system; the lymphatics, etc., etc.; nervous sympathy, direct, remote and contiguous; the functions of the various organs and tissues; the effect of mind over matter; the process of digestion and assimilation; secretion and excretion; the occult process of making blood, etc., etc. In fact, he must become thoroughly familiar with the whole science of physiology, and when he has mastered this, and thoroughly knows the human body in its healthy normal or physiological state, then, and not until then, is he prepared to study it in its diseased or pathological condition.

Thus prepared, he is now to learn the departures from a healthy to a diseased condition, and these departures are protean, presenting so wide a field that well may he tremble when he undertakes to unravel its meshes. In this department the best thought of all the ages has been expended. All the collateral sciences, and almost every science, has been made tributary to the elucidation of this most difficult science.

Chemistry, in its grand achievements, has been made votary to the clearing away of the mists. Electricity has been brought in to render assistance, while the magnifying lense has been made to contribute its wonderful power to aid in the research after truth, bringing to light many of the hidden causes of disease, and giving to the earnest searcher after truth grand results, which have in the last few years changed the whole theory of the etiology of disease. By the discovery of the science of etiology the medical profession made an advance step in the science of medicine which distanced all the former ages. Out of this discovery has come that which may well be termed the science of preventive medicine—asepsis, antisepsis, sanitation and hygiene. The doctor must be familiar with all these departments of medical science, and so understand them that he may utilize them and make them subservient to his will.

The doctor must be educated in all that pertains to his profession, that he may never be at a loss for a good and sufficient reason for his faith.

I might next allude to therapie, or therapeutics—the science and art of healing. This is the most essential part of medical science, while all the other departments are absolutely necessary, and the doctor must thoroughly understand them ; yet he may be master of all these, and without a knowledge of therapie he would be helpless to accomplish good. In order, therefore, to be competent to meet the obligations that are upon him, and meet and combat morbid conditions, the doctor must first know the nature of the disease ; in order to know this, he must have a knowledge of anatomy, physiology and pathology, and he must know the various physical signs that are present in both health and disease, so that by classifying, comparing and grouping them, he may correctly diagnose the disease, and when he has made a correct diagnosis, then he must know the therapeutic properties or action of the remedies he will use. When these are known and properly applied, therapeutics becomes a grand and glorious science. In the department of materia medica and therapeutics, the profession has always had earnest workers in their ranks, and like the busy bee, they have culled not only from every flower, but they have carried their researches throughout the organic and inorganic kingdoms until, as a result of scientific research and labor, at the present time the profession is ready to cry hold! enough. But notwithstanding all this labor and scientific research, there are still morbid conditions that we are not yet able to heal by any known remedy. I need not allude in this paper to the various other sciences or branches of the profession of medicine that the student must master, before he can be a doctor. But some may say that the specialist need not master all these departments of the science of medicine. I tell you that no man can be a specialist without a knowledge of medicine in all of its essential departments. The surgeon, the oculist, etc., must not only understand anatomy, but he must be thoroughly up in physiology, pathology and therapeutics. If he would be a doctor in the true sense of the term he must know the entire curriculum of medical science. Now, think you, my hearers, that a man can ever accomplish all this unless he had a mind well trained and that mind well in hand? Do you imagine for a moment that a man whose mind is not educated can ever master the various sciences that go in to make up the term medical science; that a man whose mind is untrained to think can ever meet the great demands of a conscious, manly uprightness when he goes before the world and asks them to trust him with the lives of those dearest to them on earth, and at the time he knows that he is unable and incompetent to understand or comprehend the simplest principles of medical philosophy? I take the ground boldly that this great and learned profession, of which we here to-day are so proud, is suffering materially, and that the public is suffering, and that it is no wonder that the medical profession received no more honor and respect in this country of ours. Why? The answer is plain enough. The profession has been subsidized by utilitarianism. Too many in the profession are using it alone for the gain that is in it. The idea now prevails with too many young men who are anxious to enter the learned profession of medicine, to make money, and they are so eager in this fast age that they are not willing to take the time to fit themselves to enter the learned profession. It has been truly said that "in education the usefulness of a study is not to be measured by its availability for the business purposes of life, but solely by its fitness to develope or educate the mental powers." The object of education is not to learn useful things, but to become able to learn how to use them, and it is a great wrong to the medical student for him to be possessed with the idea of mere money-making as the stimulant to his studies; that man is a failure as a doctor from the start, in the true sense of that term. The allurements of mammon, or the glamour of of prospective riches too often attract our young men from pursuing a thorough course of study and fitting themselves for honorable places in the profession, and by this neglect they are dwarfed and lost to the profession and the world. In this fast age short roads, near cuts and by-paths through cheap medical schools are opened up to tempt young men to abandon the proper work of thorough preparation for successful professional life work, and they hurry into these professonal schools prematurely and without preparation.

The consequence is the sending forth of half educated men and inexperienced and incompetent men to attempt to heal disease, to meet the great responsibilities of life. Let us, as members of the Tri-State Medical Association, protest against this great evil. Let us lift the standard of professional education by our voice in a solid front against this great wrong to the profession, and greater wrong to the public; and let us not hold our peace until the cheap medical school is dethroned and blotted out from our land. I mean by this the school that permits men to pass out with diplomas, and these men, many of them, without the commonest rudiments of even an English education. Let our colleges, universities, academies, etc., educate and make men first, and then let our medical schools make doctors afterwards. The time has come in the medical profession when the demand for primary education for this learned profession must be heeded, and I want the approval to be voiced by this Association, so that we may be in line with the advanced thought of the age.

In order that a man may be a doctor, he must not only be educated, but he must of necessity be a gentleman, and no man is a doctor in the true sense of that term who does not towards his professional brethren observe the golden rule—do unto others as you would that they should do unto you. To the doctor is confided the most sacred confidences of families and individuals; these must be locked within his own bosom, inviolate.

The doctor ought, to say the least, be a moral man. Temperate habits is an indispensable qualification.

To be a doctor, then, in the full meaning of the title, the man must be well educated, that is, the mental faculties must be fully developed and trained for systematic thinking; he must be familiar with the various sciences that go to make up the *reportoire* of the profession; he must be a gentleman, be temperate, moral and honest in all the relations of life.

Such a man, or such a doctor, is a man honored and esteemed by all, and no grander character can be found in all this earth. The highest type of a man, the grandest character among men, is the man who sacrifices self for the good of others. 'Tis said of the son of man when on the earth that he went about doing good. The earnest, conscientious, competent doctor comes nearest fulfilling this and following this grand example, when in the full discharge of his duties and obligations. His greatest delight is in relieving distress, alleviating pain, triumphing over disease, bringing gladness and joy into families and communities by the successful application of the principles of medical science to diseased conditions of the human family.

I congratulate you to-day, my professional fraters, because of your portentious environments. We to-day, the last decade of the nineteenth century, stand on the isthmus between the past and future, and from this proud eminence, aided by all the light and learning of the past ages, by the erudition of all the grand masters that have come and gone; aided by all the learning of this present day, and from the behest of all the ages past, gather wisdom for the future. Where much is given, much is required. I congratulate you that with these advantages, notwithstanding, your responsibilities are increased, nevertheless, your opportunities to meet these responsibilities are greatly enhanced, and while I congratulate you upon your achievements in the past, would urge upon you increased devotion to the sacred trust committed to you in the years to come.

